# QSPR analysis of distance-based structural indices for drug compounds in tuberculosis treatment

**DOI:** 10.1016/j.heliyon.2024.e23981

**Published:** 2024-01-08

**Authors:** Micheal Arockiaraj, Joseph H. Campena, A. Berin Greeni, Muhammad Usman Ghani, S. Gajavalli, Fairouz Tchier, Ahmad Zubair Jan

**Affiliations:** a Department of Mathematics, Loyola College, Chennai 600034, India; b Department of Mathematics and Statistics, College of Science, De La Salle University, 2401 Taft Avenue, Malate, Manila, 1004 Metro Manila, Philippines; c School of Advanced Sciences, Vellore Institute of Technology, Chennai 600127, India; d Institute of Mathematics, Khawaja Fareed University of Engineering Information Technology, Abu Dhabi Road, 64200, Rahim Yar Khan, Pakistan; e Mathematics Department, King Saudi University, Riyadh, 145111, Saudi Arabia; f Wroclaw University of Science and Technology, Faculty of Mechanical Engineering, Poland

**Keywords:** QSPR, Physicochemical properties, Anti-tuberculosis drugs, Topological indices

## Abstract

Tuberculosis (TB) is one of the most contagious diseases that has a greater mortality rate than HIV/AIDS and the cases of TB are feared to rise as a repercussion of the COVID-19 pandemic. The pharmaceutical industry is constantly looking for ways to improve drug design processes in order to combat the growth of infections and cure newly identified syndromes or genetically based dysfunctions with the help of QSPR models. QSPR models are mathematical tools that establish relationships between a molecular structure and its physicochemical attributes using structural properties. Topological indices are such properties that are generated from the molecular graph without any empirically derived measurements. This work focuses on developing a QSPR model using distance-based topological indices for anti-tuberculosis medications and their diverse physicochemical features.

## 1 Introduction

Chemical graph theory is a powerful and versatile field that has wide-ranging applications in various areas of chemistry and materials science. Its importance lies in providing a mathematical framework for understanding the structural properties and behaviors of chemical compounds, which can be leveraged for drug discovery, materials design, computational chemistry, and chemical informatics. Topological indices are mathematical quantities used in chemical graph theory that have significant interest owing to their applications in QSPR/QSAR investigations, which favor forecasting the potency of a drug candidate. Topological indices are an efficient way to convert chemical composition into numerical values that can be utilized for correlating with physical attributes [1], [2], [3], [4]. There have been numerous topological indices investigated so far in which most useful topological indices are based on distance or degree of molecular compounds. In 1947, Wiener proposed the first distance-based index which was later renamed as Wiener index and that study demonstrated a strong correlation between the graph theoretical parameters and the boiling point of alkanes. This paved the way for the advent of distance type topological indices.

In our study, we discuss the distance-based indices including vertex and edge versions of Wiener, Szeged, Mostar and PI. Though the mathematical investigation of the Wiener index started in the 1970s, its edge-version was brought to light only in 2009 [5]. Gutman described the Szeged index in 1994 as a mechanism for calculating the Wiener index of trees and extending it to cyclic molecular structures [6]. In 2008, Gutman and Ashrafi proposed the edge category of the Szeged index [7] and the PI index was introduced by Khadikar et al. [8] with its diverse applications. The Mostar index was popularized by Doslic et al. [9] in 2018 and its edge version was put forward by Arockiaraj et al. [10] with their suggested methods for computing the Mostar type indices.

Mycobacterium tuberculosis is the bacterium that causes the contagious airborne illness known as tuberculosis. Although it is a disease that frequently affects the lungs, it can spread to almost every part of the body. On average, a third of the world's population is believed to have been infected with TB. Even though tuberculosis is often treated with medicine, in some circumstances, particularly when there is drug resistance and certain pulmonary tuberculosis complications, it may even be treated surgically [11]. Tuberculosis remains a top infectious killer of humans [12]. Nearly all tuberculosis infections are asymptomatic and remain latent, despite the fact that some people who acquire them go on to have active tuberculosis disease [13]. Reports suggest that predominantly men develop the disease more than women. According to the literature, HIV-positive individuals are highly prone to active TB disease. Following the Declaration of Cape Town in 2000, there has been a boom in interest in anti-tuberculosis medications, raising the possibility that far less harmful, shorter regimens of curative treatment may be developed [14]. Amikacin, bedaquiline, clofazimine, imipenem-cilastatin, linezolid, moxifloxacin, terizidone, ethambutol, ethionamide, isoniazid, levofloxacin, *p*-aminosalicylic acid, and pyrazinamide are the medications frequently prescribed to treat TB [15]. Since tuberculosis is treatable, it is worth noting that there is presently no vaccine available to successfully prevent tuberculosis in adults [16]. Hence, tuberculosis is unlikely to be eradicated without improved vaccinations [17].

Aminoglycosides are a group of antibiotics used since the 1940s to primarily treat a broad spectrum of bacterial infections [18]. Bedaquiline acts as an inevitable drug in the treatment of multidrug resistant tuberculosis [19]. Clofazimine is a riminophenazine dye-based fat-soluble antibiotic with antimycobacterial and anti-inflammatory properties. Clofazimine was initially developed as an anti-tuberculosis medication, but it was later used to treat leprosy. More recently, it has been demonstrated that clofazimine can be utilized in the treatment of hospitalised COVID-19 patients. It is anticipated that it will play a part in containing any potential coronavirus diseases in the future [20].

Ethambutol, ethionamide and linezolid are important anti-tuberculosis drugs used in the management of multi-drug resistant tuberculosis [21], [22], [23]. Imipenem-cilastatin is a drug used in group C medication [24]. Isoniazid is a drug recommended for the treatment of latent tuberculosis infection [25]. Levofloxacin was chosen as a versatile antibiotic that works on both Gram-positive and Gram-negative bacteria [26]. A key medication for treating a variety of diseases is the respiratory quinolone moxifloxacin (MOX), particularly for the treatment of tuberculosis and pneumonia [27], [28].

Numerous degree-based indices for the TB drugs were computed with QSPR analysis in [29] and computation of polynomials in [30], [31]. On the other side, a lot of research has been devoted to COVID-19 drugs by considering different graph theoretical parameters induced by degree and its variations [32], [33], [34], [35] and for cancer drugs [36], [37]. In this paper, we aim to obtain a QSPR model between a few distance-based topological indices including Wiener, Szeged, PI and Mostar for the above described TB drugs and their physicochemical features. In the process of computing the indices we have employed the cut technique which involves identifying appropriate edge cuts that disintegrate the drug compound into many components that are convex and then the required property of the graph is derived from the properties of the components.

## 2 Topological indices

In this section, several basic graph theoretic definitions and key concepts required for the cut method are presented [38], [39], [40], [41], [42]. Throughout this paper, molecular graphs that must be connected are taken into account and represented as *G*. The set V(G) contains all vertices of *G* whereas E(G) with all edges. For s∈V(G), $p=s_{1}s_{2}\in E(G)$ and $q=s_{3}s_{4}\in E(G)$, the length of the shortest path between *s* and $s_{1}$ is called vertex-vertex distance and denoted by $d_{G}(s,s_{1})$. The vertex-edge distance between *s* and *p*, $d_{G}(s,p)$, is calculated by taking the smallest value between $d_{G}(s,s_{1})$ and $d_{G}(s,s_{2})$ whereas the edge-edge distance between *p* and *q*, $D_{G}(p,q)$, is measured by taking the smallest value between $d_{G}(p,s_{3})$ and $d_{G}(p,s_{4})$.

A subgraph $G_{1}$ of *G* is said to be an isometric subgraph if $d_{G_{1}}(s_{1},s_{2})$ = $d_{G}(s_{1},s_{2})$ for all $s_{1},s_{2}\in V(G_{1})$. On the other hand, $G_{1}$ is called a convex subgraph if for every pair of vertices in $G_{1}$, each shortest path between them lies completely in $G_{1}$. An important class of molecular structures, the partial cube, can be identified as an isometric subgraph of a hypercube, where the hypercube is defined by binary coordinates and edges connecting vertices that differ in one coordinate. The Djoković-Winkler relation referred to as relation Θ for two edges $p=s_{1}s_{2}$ and $q=s_{3}s_{4}$ whenever $d_{G}(s_{1},s_{3})+d_{G}(s_{2},s_{4})\neq d_{G}(s_{1},s_{4})+d_{G}(s_{2},s_{3})$ holds. The relation Θ is an equivalence relation for molecular compounds induced from partial cubes but in general its transitive closure $\Theta^{⁎}$ is an equivalence relation. Let the $\Theta^{⁎}$-partition of *G* be P = $\left\{S_{1},S_{2},\ldots ,S_{k}\right\}$. The quotient graph $G/S_{i}$, for any class $S_{i}$ is obtained through the disconnected graph $G-S_{i}$, the vertices being the connected components. A component $X_{m}^{i}$ is adjacent to a component $X_{n}^{i}$, if a vertex *u* in component $X_{m}^{i}$ is adjacent to a vertex *v* in component $X_{n}^{i}$ with $uv\in S_{i}$.

For $p=s_{1}s_{2}\in E(G)$, the sets $N_{s_{1}}(p|G)$ and $M_{s_{1}}(p|G)$ are characterized as $N_{s_{1}}(p|G)$ = $\left\{s\in V(G):d_{G}(s_{1},s)<d_{G}(s_{2},s)\right\}$ and $M_{s_{1}}(p|G)$ = $\left\{e\in E(G):d_{G}(s_{1},e)<d_{G}(s_{2},e)\right\}$ respectively. The cardinality of the above mentioned sets are represented as $n_{s_{1}}(p|G)$ and $m_{s_{1}}(p|G)$ respectively. The quantities $n_{s_{2}}(p|G)$ and $m_{s_{2}}(p|G)$ are analogous.

The notation of strength-weighted graph was initially introduced by Arockiaraj et al. [43] for computing the topological indices. It is denoted as $G_{sw}$ = $\left(G,(w_{a},s_{a}),s_{b}\right)$ where, $w_{a},s_{a}$ and $s_{b}$ represent the weight of the vertex, strength of the vertex and strength of the edge respectively. The distance functions for a strength-weighted graph are calculated by considering the underlying simple graph *G*. The sets $N_{s_{1}}(p|G_{sw})$ and $M_{s_{1}}(p|G_{sw})$ are similar to the sets $N_{s_{1}}(p|G)$ and $M_{s_{1}}(p|G)$ respectively, but their cardinalities are described as

$$n_{s_{1}}(p|G_{sw})=\sum_{x\in N_{s_{1}}(p|G_{sw})}w_{a}(x)\;\text{and}\;m_{s_{1}}(p|G_{sw})=\sum_{x\in N_{s_{1}}(p|G_{sw})}s_{a}(x)+\sum_{y\in M_{s_{1}}(p|G_{sw})}s_{b}(y).$$

The sets $N_{s_{2}}(p|G_{sw})$ and $M_{s_{2}}(p|G_{sw})$ and their respective cardinalities can be characterized similarly.

The topological index (TI) of a strength-weighted graph $G_{sw}$ can be reduced to a simple graph *G* whenever $w_{a}$ = 1, $s_{a}$ = 0 and $s_{b}$ = 1. The following expressions pertaining to $G_{sw}$ act as the tool for the derivation of the topological indices, which are the vertex and edge versions of Wiener (*W* and $W_{e}$), Szeged ($Sz_{v}$ and $Sz_{e}$), Mostar (*Mo* and $Mo_{e}$) and Padmakar-Ivan (*PI*) [10], [43], [44].

(1) $$W(G_{sw})=\sum_{\left\{s_{1},s_{2}\}\subseteq V(G_{sw}\right)}w_{a}(s_{1})w_{a}(s_{2})d_{G_{sw}}(s_{1},s_{2})$$

(2) $$W_{e}(G_{sw})=\sum_{\left\{s_{1},s_{2}\}\subseteq V(G_{sw}\right)}s_{a}(s_{1})s_{a}(s_{2})d_{G_{sw}}(s_{1},s_{2})+\sum_{\left\{p,q\}\subseteq E(G_{sw}\right)}s_{b}(p)s_{b}(q)D_{G_{sw}}(p,q)+\sum_{s\in V(G_{sw})}\sum_{p\in E(G_{sw})}s_{a}(s)s_{b}(p)d_{G_{sw}}(s,p)$$

(3) $$Sz_{v}(G_{sw})=\sum_{p=s_{1}s_{2}\in E(G_{sw})}s_{b}(p)n_{s_{1}}(p|G_{sw})n_{s_{2}}(p|G_{sw})$$

(4) $$Sz_{e}(G_{sw})=\sum_{p=s_{1}s_{2}\in E(G_{sw})}s_{b}(p)m_{s_{1}}(p|G_{sw})m_{s_{2}}(p|G_{sw})$$

(5) $$PI(G_{sw})=\sum_{p=s_{1}s_{2}\in E(G_{sw})}s_{b}(p)[m_{s_{1}}(p|G_{sw})+m_{s_{2}}(p|G_{sw})]$$

(6) $$Mo(G_{sw})=\sum_{p=s_{1}s_{2}\in E(G_{sw})}s_{b}(p)|n_{s_{1}}(p|G_{sw})-n_{s_{2}}(p|G_{sw})|$$

(7) $$Mo_{e}(G_{sw})=\sum_{p=s_{1}s_{2}\in E(G_{sw})}s_{b}(p)|m_{s_{1}}(p|G_{sw})-m_{s_{2}}(p|G_{sw})|$$

## 3 Methodology

To compute the distance based topological indices of novel drug compounds used in the treatment of tuberculosis, we enumerate the bonds of drug compounds based on the Θ-relation and derive the graph theoretical parameters. Such parameters are used to find the numerical quantities for vertex and edge versions of Wiener, Szeged, Mostar and PI indices by the mathematical method described below.

Theorem 3.1 [10], [43], [44]*For*$TI\in \{W,W_{e},SZ_{v},SZ_{e},PI,MO,MO_{e}\}$*, let*P*=*$\left\{S_{1},S_{2},\ldots ,S_{k}\right\}$*be the*$\Theta^{⁎}$*-partition of*$G_{sw}$*=*$\left(G,(w_{a},s_{a}),s_{b}\right)$*. Then,*$TI(G_{sw})$*=*$\sum_{i=1}^{k}TI(G/S_{i},(w_{a}^{i},s_{a}^{i}),s_{b}^{i})$*where*

• $w_{a}^{i}$*:*$V(G/S_{i})\to R_{0}^{+}$*is defined by*$w_{a}^{i}(X)$*=*$\sum_{x\in V(X)}w_{a}(x)$*, for all connected components*$X\in G/S_{i}$*,*
• $s_{a}^{i}$*:*$V(G/S_{i})\to R_{0}^{+}$*is defined by*$s_{a}^{i}(X)$*=*$\sum_{xy\in E(X)}s_{b}(xy)$*+*$\sum_{x\in V(X)}s_{a}(x)$*, for all connected components*$X\in G/S_{i}$*,*
• $s_{b}^{i}$*:*$E(G/S_{i})\to R_{0}^{+}$*is defined by*$s_{b}^{i}(XY)=|\{xy\in S_{i}:x\in X,y\in Y\}|$*for all pairs of components X and Y of*$G/S_{i}$*.*

**Figure 1:**
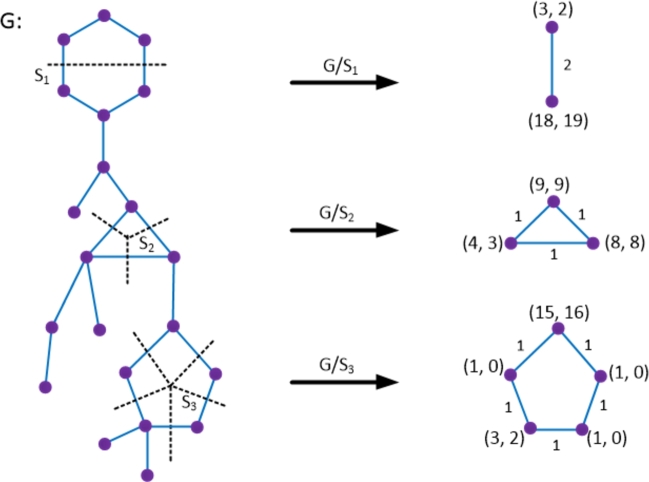
Illustration for determining quotient graphs induced from Θ^⁎^-classes.

**Figure 2:**
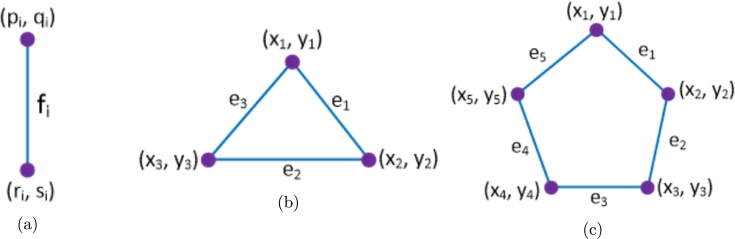
Describing the functions for vertex weight, vertex strength, and edge strength in the context of specific quotient graphs (a) edge (b) triangle (c) pentagon.

To demonstrate the aforementioned approach, we examine a graph *G*, as depicted in Fig. 1. From this graph, we extract the quotient graphs for the Θ-class $S_{1}$ and the $\Theta^{⁎}$-classes $S_{2}$ and $S_{3}$, which correspond to a single edge, a triangle, and a pentagon, respectively. The determination of the vertex weight and strength function $\left(w_{a}^{i},s_{a}^{i}\right)$ and the edge strength function $s_{b}^{i}$ is accomplished by counting the vertices and edges within the connected components of the quotient graphs, along with the edges within the $\Theta^{⁎}$-classes as illustrated in Fig. 1. In the scope of our current research, we have the quotient graphs showcased in Fig. 1. Consequently, we provide a detailed description of the strength-weighted functions, as presented in Fig. 2(a–c), to streamline our computational procedures.

In a general context, considering any Θ-class $S_{i}$, we define $\left(p_{i},q_{i}\right)$ and $\left(r_{i},s_{i}\right)$ as the vertex strength-weighted values for two components resulting from the removal of $S_{i}$. Additionally, we denote the edge strength as $f_{i}$, with $p_{i}+r_{i}=|V(G)|$ and $q_{i}+s_{i}+f_{i}=|E(G)|$. In the context of a particular $\Theta^{⁎}$-class leading to a triangle, we specify the vertex strength-weighted values as $\left(x_{i},y_{i}\right)$ and the edge strength as $e_{i}$ for 1≤i≤3 in which $x_{1}+x_{2}+x_{3}=|V(G)|$ and $y_{1}+y_{2}+y_{3}+e_{1}+e_{2}+e_{3}=|E(G)|$. Similarly, for the case of a pentagon, the values are denoted as $\left(x_{i},y_{i}\right)$ and $e_{i}$ for 1≤i≤5 where $x_{1}+...+x_{5}=|V(G)|$ and $y_{1}+...+y_{5}+e_{1}+...+e_{5}=|E(G)|$. We utilize these graph theoretical parameters as illustrated in Fig. 2(a–c) to compute topological indices, which are subsequently compared with the physicochemical attributes of anti-TB drugs. This comparison facilitates the development of quantitative structure-property relationship (QSPR) models.

## 4 Results

We denote the TB drug compounds amikacin, bedaquiline, clofazimine, imipenem-cilastatin, linezolid, moxifloxacin, terizidone, ethambutol, ethionamide, isoniazid, levofloxacin, *p*-aminosalicylic acid, and pyrazinamide as $D_{1}$, $D_{2}$, $D_{3}$, …, $D_{13}$ respectively.

Result 4.1 Let $D_{1}$ be an amikacin compound. Then $W(D_{1})$ = 5207, $W_{e}(D_{1})$ = 4352, $SZ_{v}(D_{1})$ = 7596, $SZ_{e}(D_{1})$ = 6554, $PI(D_{1})$ = 1704, $MO(D_{1})$ = 1172 and $MO_{e}(D_{1})$ = 1264.

**Figure 3:**
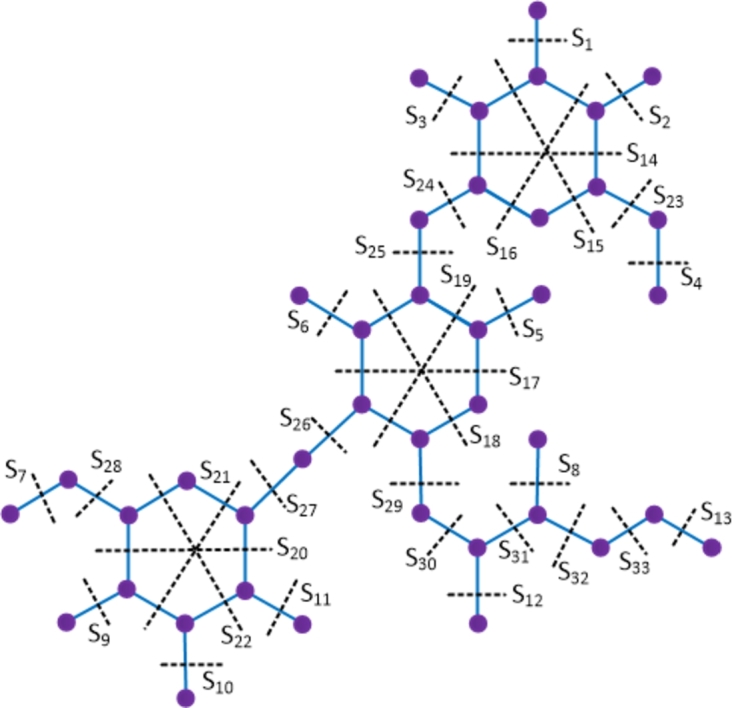
Enumeration of Θ-classes *S*_*i*_ in the amikacin drug.

Proof The amikacin compound has 40 vertices and 42 edges. Let $\{S_{i}$: 1≤i≤33} be the Θ cuts of $D_{1}$ which partition the compound into 2 convex components. The molecular graph of the amikacin compound along with the Θ cuts is depicted in Fig. 3 and strength-weighted quantities are presented in Table 1.

**Table 1:** Strength-weighted quantities of *D*_1_/*S*_*i*_.

***S***_***i***_	**1 ≤ *i* ≤ 13**	**14**	**15**	**16**	**17**	**18**	**19**	**20**	**21**	**22**
***p***_***i***_	1	6	7	6	17	16	12	6	6	7
***q***_***i***_	0	5	6	5	17	16	11	5	5	6
***f***_***i***_	1	2	2	2	2	2	2	2	2	2



***S***_***i***_	**23**	**24**	**25**	**26**	**27**	**28**	**29**	**30**	**31**	**32**	**33**
***p***_***i***_	2	11	12	12	11	2	8	7	5	3	2
***q***_***i***_	1	11	12	12	11	1	7	6	4	2	1
***f***_***i***_	1	1	1	1	1	1	1	1	1	1	1

Using the Eqs. (1) − (7) in Theorem 3.1, the topological indices are computed as $W(D_{1})$ = $\sum_{i=1}^{33}p_{i}r_{i}$ = 5207, $W_{e}(D_{1})$ = $\sum_{i=1}^{33}q_{i}s_{i}$ = 4352, $Sz_{v}(D_{1})$ = $\sum_{i=1}^{33}f_{i}p_{i}r_{i}$ = 7596, $Sz_{e}(D_{1})$ = $\sum_{i=1}^{33}f_{i}q_{i}s_{i}$ = 6554, $PI(D_{1})$ = $\sum_{i=1}^{33}f_{i}(q_{i}+s_{i}$) = 1704, $Mo(D_{1})$ = $\sum_{i=1}^{33}f_{i}|p_{i}-r_{i}|$ = 1172 and $Mo_{e}(D_{1})$ = $\sum_{i=1}^{33}f_{i}|q_{i}-s_{i}|$ = 1264. □

Result 4.2 Let $D_{2}$ be a bedaquiline compound. Then $W(D_{2})$ = 3672, $W_{e}(D_{2})$ = 3361, $SZ_{v}(D_{2})$ = 5962, $SZ_{e}(D_{2})$ = 5397, $PI(D_{2})$ = 1606, $MO(D_{2})$ = 1119 and $MO_{e}(D_{2})$ = 1264.

**Figure 4:**
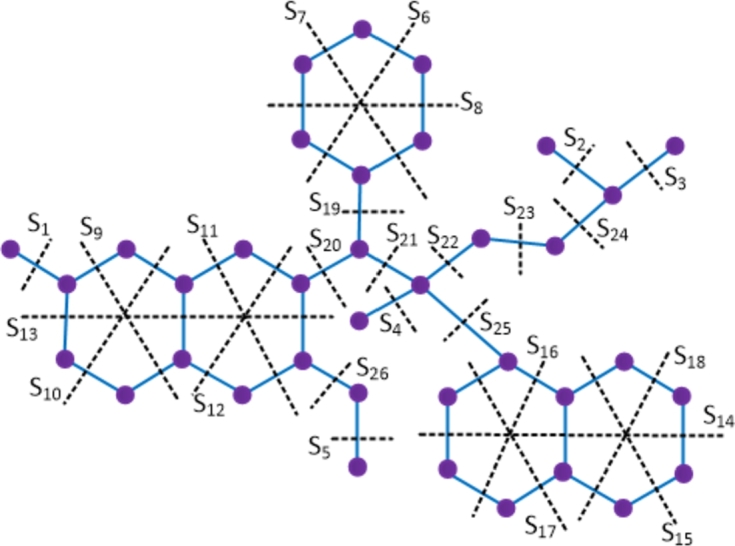
Enumeration of Θ-classes *S*_*i*_ in the bedaquiline drug.

Proof The bedaquiline compound has 37 vertices and 41 edges. Let $\{S_{i}$: 1≤i≤26} be the Θ cuts of $D_{2}$. The molecular graph of the bedaquiline compound along with the Θ cuts is depicted in Fig. 4 and the quantities of quotient graphs are presented in Table 2.

**Table 2:** Strength-weighted quantities of *D*_2_/*S*_*i*_.

***S***_***i***_	**1 ≤ *i* ≤ 5**	***i* = 6,7,8**	***i* = 9,10**	***i* = 11,12**	**13**	**14**	**15**
***p***_***i***_	1	3	4	8	7	5	3
***q***_***i***_	0	2	3	8	6	4	2
***f***_***i***_	1	2	2	2	3	3	2



***S***_***i***_	**16**	***i* = 17,18**	**19**	**20**	**21**	**22**	**23**	**24**	**25**	**26**
***p***_***i***_	7	3	6	13	17	5	4	3	10	2
***q***_***i***_	7	2	6	14	18	4	3	2	11	1
***f***_***i***_	2	2	1	1	1	1	1	1	1	1

Then, using the Eqs. (1) − (7) in Theorem 3.1, the topological indices are computed as $W(D_{2})$ = $\sum_{i=1}^{26}p_{i}r_{i}$ = 3672, $W_{e}(D_{2})$ = $\sum_{i=1}^{26}q_{i}s_{i}$ = 3361, $Sz_{v}(D_{2})$ = $\sum_{i=1}^{26}f_{i}p_{i}r_{i}$ = 5962, $Sz_{e}(D_{2})$ = $\sum_{i=1}^{26}f_{i}q_{i}s_{i}$ = 5397, $PI(D_{2})$ = $\sum_{i=1}^{26}f_{i}(q_{i}+s_{i}$) = 1606, $Mo(D_{2})$ = $\sum_{i=1}^{26}f_{i}|p_{i}-r_{i}|$ = 1119 and $Mo_{e}(D_{2})$ = $\sum_{i=1}^{26}f_{i}|q_{i}-s_{i}|$ = 1264. □

Result 4.3 Let $D_{3}$ be a clofazimine compound. Then $W(D_{3})$ = 3032, $W_{e}(D_{3})$ = 2865, $SZ_{v}(D_{3})$ = 5706, $SZ_{e}(D_{3})$ = 5485, $PI(D_{3})$ = 1296, $MO(D_{3})$ = 711 and $MO_{e}(D_{3})$ = 826.

**Figure 5:**
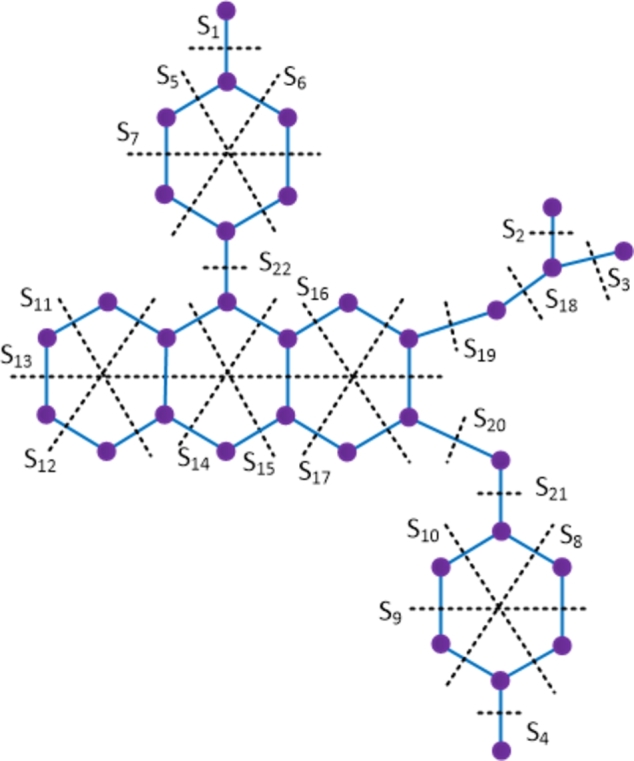
Enumeration of Θ-classes *S*_*i*_ in the clofazimine drug.

Proof The clofazimine compound has 33 vertices and 37 edges. Let $\{S_{i}$: 1≤i≤22} be the Θ cuts of $D_{3}$. The molecular graph of the clofazimine compound along with the Θ cuts is depicted in Fig. 5 and the parameters of quotient graphs are presented in Table 3. Using the Eqs. (1) − (7) in Theorem 3.1, the topological indices are computed as $W(D_{3})$ = $\sum_{i=1}^{22}p_{i}r_{i}$ = 3032, $W_{e}(D_{3})$ = $\sum_{i=1}^{22}q_{i}s_{i}$ = 2865, $Sz_{v}(D_{3})$ = $\sum_{i=1}^{22}f_{i}p_{i}r_{i}$ = 5706, $Sz_{e}(D_{3})$ = $\sum_{i=1}^{22}f_{i}q_{i}s_{i}$ = 5485, $PI(D_{3})$ = $\sum_{i=1}^{22}f_{i}(q_{i}+s_{i}$) = 1296, $Mo(D_{3})$ = $\sum_{i=1}^{22}f_{i}|p_{i}-r_{i}|$ = 711 and $Mo_{e}(D_{3})$ = $\sum_{i=1}^{22}f_{i}|q_{i}-s_{i}|$ = 826. □

**Table 3:** Strength-weighted quantities of *D*_3_/*S*_*i*_.

***S***_***i***_	**1 ≤ *i* ≤ 4**	**5 ≤ *i* ≤ 10**	***i* = 11,12**	**13**	**14**	**15**
***p***_***i***_	1	4	3	15	14	7
***q***_***i***_	0	3	2	15	15	7
***f***_***i***_	1	2	2	4	2	2



***S***_***i***_	***i* = 16,17**	**18**	**19**	**20**	***i* = 21,22**
***p***_***i***_	15	3	4	8	7
***q***_***i***_	15	2	3	8	7
***f***_***i***_	2	1	1	1	1

Result 4.4 Let $D_{4}$ be an imipenem-cilastatin compound. Then $W(D_{4})$ = 843, $W_{e}(D_{4})$ = 597, $SZ_{v}(D_{4})$ = 1052, $SZ_{e}(D_{4})$ = 853, $PI(D_{4})$ = 395, $MO(D_{4})$ = 223 and $MO_{e}(D_{4})$ = 249.

**Figure 6:**
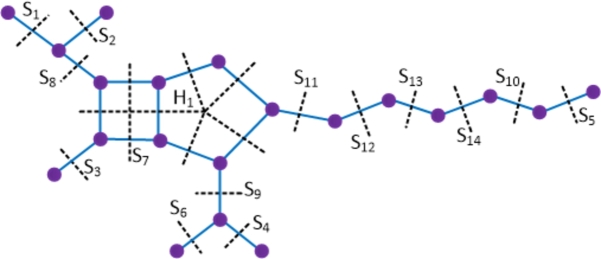
Enumeration of Θ^⁎^-classes in the imipenem-cilastatin drug.

Proof With 20 vertices and 21 edges, the molecular graph of imipenem-cilastatin compound has a pentagon and a $C_{4}$ sharing a common edge and hence imipenem-cilastatin is not a partial cube. Therefore to evaluate the mentioned topological indices, we employ the $\Theta^{⁎}$-partition. Let $\left\{S_{i}:1\leq i\leq 14\}⋃\{H_{1}\right\}$ be the $\Theta^{⁎}$-partition of $D_{4}$. The molecular graph of the imipenem-cilastatin compound along with the $\Theta^{⁎}$ cuts is depicted in Fig. 6. Strength-weighted quantities of the quotient graphs $D_{4}/S_{i}$ and $D_{4}/H_{1}$ are presented in Table 4, Table 5 respectively.

**Table 4:** Strength-weighted quantities of *D*_4_/*S*_*i*_.

***S***_***i***_	**1 ≤ *i* ≤ 6**	**7**	***i* = 8,9**	**10**	**11**	**12**	**13**	**14**
***p***_***i***_	1	6	3	18	14	15	16	17
***q***_***i***_	0	5	2	19	15	16	17	18
***f***_***i***_	1	2	1	1	1	1	1	1

**Table 5:** Strength-weighted values of *D*_4_/*H*_1_.

j	**1**	**2**	**3**	**4**	**5**
***x***_***j***_	1	7	4	3	5
***y***_***j***_	0	6	3	2	4
***e***_***j***_	1	1	1	2	1

Due to the presence of a pentagon in the drug compound $D_{4}$, the reduced quotient graph resulting from $\Theta^{⁎}$ covering the pentagon's edges corresponds to the pentagon structure itself. Therefore, the computation of topological indices is a bit involved and deduced from the primary definitions of topological indices in the Eqs. (1) − (7) and using Theorem 3.1, which is explained below.

$$W(D_{4})=\sum_{i=1}^{14}p_{i}r_{i}+x_{1}(x_{2}+2x_{3}+2x_{4}+x_{5})+x_{2}(x_{3}+2x_{4}+2x_{5})+x_{3}(x_{4}+2x_{5})+x_{4}x_{5}=843.W_{e}(D_{4})=\sum_{i=1}^{14}q_{i}s_{i}+y_{2}y_{3}+2y_{2}y_{4}+2y_{2}y_{5}+y_{3}y_{4}+2y_{3}y_{5}+y_{4}y_{5}+e_{1}(e_{3}+e_{4})+e_{2}(e_{4}+e_{5})+e_{3}e_{5}+y_{2}(e_{3}+2e_{4}+e_{5})+y_{3}(e_{4}+2e_{5}+e_{1})+y_{4}(e_{5}+2e_{1}+e_{2})+y_{5}(e_{1}+2e_{2}+e_{3})=597.Sz_{v}(D_{4})=\sum_{i=1}^{14}f_{i}p_{i}r_{i}+e_{1}(x_{1}+x_{5})(x_{2}+x_{3})+e_{2}(x_{2}+x_{1})(x_{3}+x_{4})+e_{3}(x_{3}+x_{2})(x_{4}+x_{5})+e_{4}(x_{5}+x_{1})(x_{4}+x_{3})+e_{5}(x_{1}+x_{2})(x_{5}+x_{4})=1052.Sz_{e}(D_{4})=\sum_{i=1}^{14}f_{i}q_{i}s_{i}+e_{1}(y_{1}+y_{5}+e_{5}+e_{4})(y_{2}+y_{3}+e_{2}+e_{3})+e_{2}(y_{2}+y_{1}+e_{1}+e_{5})(y_{3}+y_{4}+e_{3}+e_{4})+e_{3}(y_{4}+y_{5}+e_{4}+e_{5})(y_{3}+y_{2}+e_{2}+e_{1})+e_{4}(y_{5}+y_{1}+e_{5}+e_{1})(y_{4}+y_{3}+e_{3}+e_{2})+e_{5}(y_{5}+y_{4}+e_{4}+e_{3})(y_{1}+y_{2}+e_{1}+e_{2})=853.PI(D_{4})=\sum_{i=1}^{14}f_{i}(q_{i}+s_{i})+e_{1}(y_{1}+y_{5}+e_{5}+e_{4}+y_{2}+y_{3}+e_{2}+e_{3})+e_{2}(y_{2}+y_{1}+e_{1}+e_{5}+y_{3}+y_{4}+e_{3}+e_{4})+e_{3}(y_{4}+y_{5}+e_{4}+e_{5}+y_{3}+y_{2}+e_{2}+e_{1})+e_{4}(y_{5}+y_{1}+e_{5}+e_{1}+y_{4}+y_{3}+e_{3}+e_{2})+e_{5}(y_{5}+y_{4}+e_{4}+e_{3}+y_{1}+y_{2}+e_{1}+e_{2})=395.Mo(D_{4})=\sum_{i=1}^{14}f_{i}|p_{i}-r_{i}|+e_{1}|(x_{1}+x_{5})-(x_{2}+x_{3})|+e_{2}|(x_{2}+x_{1})-(x_{3}+x_{4})|+e_{3}|(x_{3}+x_{2})-(x_{4}+x_{5})|+e_{4}|(x_{5}+x_{1})-(x_{4}+x_{3})|+e_{5}|(x_{1}+x_{2})-(x_{5}+x_{4})|=223.Mo_{e}(D_{4})=\sum_{i=1}^{14}f_{i}|q_{i}-s_{i}|+e_{1}|(y_{1}+y_{5}+e_{5}+e_{4})-(y_{2}+y_{3}+e_{2}+e_{3})|+e_{2}|(y_{2}+y_{1}+e_{1}+e_{5})-(y_{3}+y_{4}+e_{3}+e_{4})|+e_{3}|(y_{4}+y_{5}+e_{4}+e_{5})-(y_{3}+y_{2}+e_{2}+e_{1})|+e_{4}|(y_{5}+y_{1}+e_{5}+e_{1})-(y_{4}+y_{3}+e_{3}+e_{2})|+e_{5}|(y_{5}+y_{4}+e_{4}+e_{3})-(y_{1}+y_{2}+e_{1}+e_{2})|=249.$$

 □

Result 4.5 Let $D_{5}$ be a linezolid compound. Then $W(D_{5})$ = 1467, $W_{e}(D_{5})$ = 1275, $SZ_{v}(D_{5})$ = 2184, $SZ_{e}(D_{5})$ = 2001, $PI(D_{5})$ = 617, $MO(D_{5})$ = 328 and $MO_{e}(D_{5})$ = 371.

**Figure 7:**
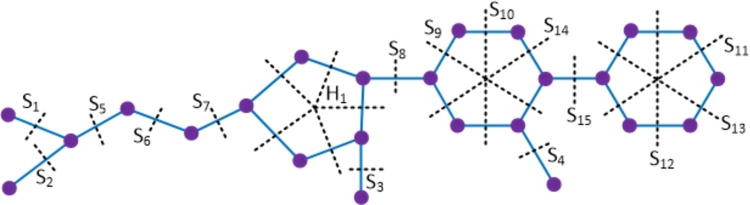
Enumeration of Θ^⁎^-classes in the linezolid drug.

Proof With 24 vertices and 26 edges, the molecular graph of linezolid compound has a pentagon and hence linezolid is not a partial cube. Therefore to evaluate the mentioned topological indices, we employ the $\Theta^{⁎}$-partition. Let $\left\{S_{i}:1\leq i\leq 15\}⋃\{H_{1}\right\}$ be the $\Theta^{⁎}$-partition of $D_{5}$. The molecular graph of the linezolid compound along with the $\Theta^{⁎}$ cuts is depicted in Fig. 7. The strength-weighted quantities of the quotient graphs $D_{5}/S_{i}$ and $D_{5}/H_{1}$ are presented in Table 6, Table 7 respectively.

**Table 6:** Strength-weighted quantities of *D*_5_/*S*_*i*_.

***S***_***i***_	**1 ≤ *i* ≤ 4**	**5**	**6**	**7**	**8**	**9**	**10**	***i* = 11,12,13**	**14**	**15**
***p***_***i***_	1	3	4	5	11	15	14	3	10	6
***q***_***i***_	0	2	3	4	11	15	14	2	10	6
***f***_***i***_	1	1	1	1	1	2	2	2	2	1

**Table 7:** Strength-weighted quantities of *D*_5_/*H*_1_.

j	**1**	**2**	**3**	**4**	**5**
***x***_***j***_	6	1	2	14	1
***y***_***j***_	5	0	1	15	0
***e***_***j***_	1	1	1	1	1

The topological indices are computed using the Eqs. (1) − (7) in Theorem 3.1 which are given below.

$$W(D_{5})=\sum_{i=1}^{15}p_{i}r_{i}+x_{1}(x_{2}+2x_{3}+2x_{4}+x_{5})+x_{2}(x_{3}+2x_{4}+2x_{5})+x_{3}(x_{4}+2x_{5})+x_{4}x_{5}=1467.W_{e}(D_{5})=\sum_{i=1}^{15}q_{i}s_{i}+(y_{1}y_{2}+2y_{1}y_{3}+2y_{1}y_{4}+y_{1}y_{5})+y_{2}y_{3}+2y_{2}y_{4}+2y_{2}y_{5}+y_{3}y_{4}+2y_{3}y_{5}+y_{4}y_{5}+e_{1}(e_{3}+e_{4})+e_{2}(e_{4}+e_{5})+e_{3}e_{5}+y_{1}(e_{2}+2e_{3}+e_{4})+y_{3}(e_{4}+2e_{5}+e_{1})+y_{4}(e_{5}+2e_{1}+e_{2})=1275.Sz_{v}(D_{5})=\sum_{i=1}^{15}f_{i}p_{i}r_{i}+e_{1}(x_{1}+x_{5})(x_{2}+x_{3})+e_{2}(x_{2}+x_{1})(x_{3}+x_{4})+e_{3}(x_{3}+x_{2})(x_{4}+x_{5})+e_{4}(x_{5}+x_{1})(x_{4}+x_{3})+e_{5}(x_{1}+x_{2})(x_{5}+x_{4})=2184.Sz_{e}(D_{5})=\sum_{i=1}^{15}f_{i}q_{i}s_{i}+e_{1}(y_{1}+y_{5}+e_{5}+e_{4})(y_{2}+y_{3}+e_{2}+e_{3})+e_{2}(y_{2}+y_{1}+e_{1}+e_{5})(y_{3}+y_{4}+e_{3}+e_{4})+e_{3}(y_{4}+y_{5}+e_{1}+e_{2})(y_{3}+y_{2}+e_{4}+e_{5})+e_{4}(y_{5}+y_{1}+e_{5}+e_{1})(y_{4}+y_{3}+e_{3}+e_{2})+e_{5}(y_{5}+y_{4}+e_{1}+e_{2})(y_{1}+y_{2}+e_{3}+e_{4})=2001.PI(D_{5})=\sum_{i=1}^{15}f_{i}(q_{i}+s_{i})+e_{1}(y_{1}+y_{5}+e_{5}+e_{4}+y_{2}+y_{3}+e_{2}+e_{3})+e_{2}(y_{2}+y_{1}+e_{1}+e_{5}+y_{3}+y_{4}+e_{3}+e_{4})+e_{3}(y_{4}+y_{5}+e_{4}+e_{5}+y_{3}+y_{2}+e_{2}+e_{1})+e_{4}(y_{5}+y_{1}+e_{5}+e_{1}+y_{4}+y_{3}+e_{3}+e_{2})+e_{5}(y_{5}+y_{4}+e_{4}+e_{3}+y_{1}+y_{2}+e_{1}+e_{2})=617.Mo(D_{5})=\sum_{i=1}^{15}f_{i}|p_{i}-r_{i}|+e_{1}|(x_{1}+x_{5})-(x_{2}+x_{3})|+e_{2}|(x_{2}+x_{1})-(x_{3}+x_{4})|+e_{3}|(x_{3}+x_{2})-(x_{4}+x_{5})|+e_{4}|(x_{5}+x_{1})-(x_{4}+x_{3})|+e_{5}|(x_{1}+x_{2})-(x_{5}+x_{4})|=328.Mo_{e}(D_{5})=\sum_{i=1}^{15}f_{i}|q_{i}-s_{i}|+e_{1}|(y_{1}+y_{5}+e_{5}+e_{4})-(y_{2}+y_{3}+e_{2}+e_{3})|+e_{2}|(y_{2}+y_{1}+e_{1}+e_{5})-(y_{4}+y_{5}+e_{3}+e_{4})|+e_{3}|(y_{2}+y_{3}+e_{4}+e_{5})-(y_{4}+y_{5}+e_{2}+e_{1})|+e_{4}|(y_{5}+y_{1}+e_{5}+e_{1})-(y_{4}+y_{3}+e_{3}+e_{2})|+e_{5}|(y_{5}+y_{4}+e_{1}+e_{2})-(y_{1}+y_{2}+e_{4}+e_{3})|=371.$$

 □

Result 4.6 Let $D_{6}$ be a moxifloxacin compound. Then $W(D_{6})$ = 2006, $W_{e}(D_{6})$ = 1908, $SZ_{v}(D_{6})$ = 3443, $SZ_{e}(D_{6})$ = 3544, $PI(D_{6})$ = 956, $MO(D_{6})$ = 516 and $MO_{e}(D_{6})$ = 608.

**Figure 8:**
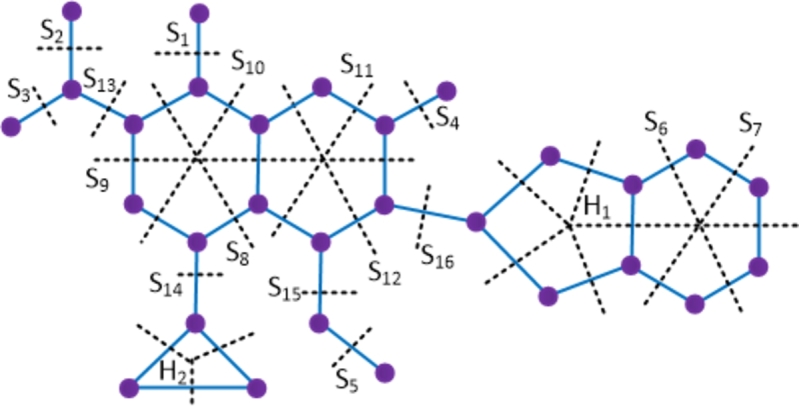
Enumeration of Θ^⁎^-classes in moxifloxacin drug.

Proof With 29 vertices and 33 edges, the molecular graph of moxifloxacin compound has a pentagon that shares an edge with a hexagon and a triangle and hence moxifloxacin is not a partial cube. Therefore to evaluate the mentioned topological indices, we employ the $\Theta^{⁎}$-partition. Let $\left\{S_{i}:1\leq i\leq 16\}⋃\{H_{1}\}⋃\{H_{2}\right\}$ be the $\Theta^{⁎}$-partition of $D_{6}$. The molecular graph of the moxifloxacin compound along with the $\Theta^{⁎}$ cuts is shown in Fig. 8. The strength-weighted quantities of the quotient graphs $D_{6}/S_{i}$ are provided in Table 8. In the case of the quotient graph $D_{6}/H_{1}$, it is a pentagon with vertex weights and strength values denoted as $\left(x_{1j},y_{1j}\right)$ and edge strength values as $e_{1j}$, 1≤j≤5. On the other hand, the quotient graph $D_{6}/H_{2}$ is a triangle, and its strength-weighted values are represented as $\left(x_{2j},y_{2j}\right)$ and $e_{2j}$, 1≤j≤3, which are provided in Table 9.

**Table 8:** Strength-weighted quantities of *D*_6_/*S*_*i*_.

***S***_***i***_	**1 ≤ *i* ≤ 5**	***i* = 6,7**	**8**	**9**	**10**	**11**	**12**	**13**	**14**	**15**	**16**
***p***_***i***_	1	3	9	10	7	14	13	3	3	2	9
***q***_***i***_	0	2	9	9	6	15	14	2	3	1	10
***f***_***i***_	1	2	2	3	2	2	2	1	1	1	1

**Table 9:** Strength-weighted quantities of *D*_6_/*H*_*k*_, *k* = 1,2.

k	***x***_***k*****1**_	***x***_***k*****2**_	***x***_***k*****3**_	***x***_***k*****4**_	***x***_***k*****5**_	***y***_***k*****1**_	***y***_***k*****2**_	***y***_***k*****3**_	***y***_***k*****4**_	***y***_***k*****5**_	***e***_***k*****1**_	***e***_***k*****2**_	***e***_***k*****3**_	***e***_***k*****4**_	***e***_***k*****5**_
**1**	1	21	1	3	3	0	23	0	2	2	1	1	1	2	1
**2**	27	1	1	-	-	30	0	0	-	-	1	1	1	-	-

Using the Eqs. (1) − (7) in Theorem 3.1, the topological indices are computed in the following.

$$W(D_{6})=\sum_{i=1}^{16}p_{i}r_{i}+x_{11}(x_{12}+2x_{13}+2x_{14}+x_{15})+x_{12}(x_{13}+2x_{14}+2x_{15})+x_{13}(x_{14}+2x_{15})+x_{14}x_{15}+x_{21}(x_{22}+x_{23})+x_{22}(x_{23})=2006.W_{e}(D_{6})=\sum_{i=1}^{16}q_{i}s_{i}+y_{12}(2y_{14}+2y_{15})+y_{14}y_{15}+e_{11}(e_{13}+e_{14})+e_{12}(e_{14}+e_{15})+e_{13}e_{15}+y_{12}(e_{13}+2e_{14}+e_{15})+y_{14}(e_{15}+2e_{11}+e_{12})+y_{15}(e_{11}+2e_{12}+e_{13})+y_{21}(e_{22})=1908.Sz_{v}(D_{6})=\sum_{i=1}^{16}f_{i}p_{i}r_{i}+e_{11}(x_{11}+x_{15})(x_{12}+x_{13})+e_{12}(x_{12}+x_{11})(x_{13}+x_{14})+e_{13}(x_{13}+x_{12})(x_{14}+x_{15})+e_{14}(x_{15}+x_{11})(x_{14}+x_{13})+e_{15}(x_{11}+x_{12})(x_{15}+x_{14})+e_{21}(x_{21}x_{22})+e_{22}(x_{22}x_{23})+e_{23}(x_{21}x_{23})=3443.Sz_{e}(D_{6})=\sum_{i=1}^{16}f_{i}q_{i}s_{i}+e_{11}(y_{11}+y_{15}+e_{15}+e_{14})(y_{12}+y_{13}+e_{12}+e_{13})+e_{12}(y_{12}+y_{11}+e_{11}+e_{15})(y_{13}+y_{14}+e_{13}+e_{14})+e_{13}(y_{14}+y_{15}+e_{14}+e_{15})(y_{13}+y_{12}+e_{12}+e_{11})+e_{14}(y_{15}+y_{11}+e_{15}+e_{11})(y_{14}+y_{13}+e_{13}+e_{12})+e_{15}(y_{15}+y_{14}+e_{14}+e_{13})(y_{11}+y_{12}+e_{11}+e_{12})+e_{21}(y_{21}+e_{23})(y_{22}+e_{22})+e_{22}(y_{22}+e_{21})(y_{23}+e_{23})+e_{23}(y_{21}+e_{21})(y_{23}+e_{22})=3544.PI(D_{6})=\sum_{i=1}^{16}f_{i}(q_{i}+s_{i})+e_{11}(y_{11}+y_{15}+e_{15}+e_{14}+y_{12}+y_{13}+e_{12}+e_{13})+e_{12}(y_{12}+y_{11}+e_{11}+e_{15}+y_{13}+y_{14}+e_{13}+e_{14})+e_{13}(y_{14}+y_{15}+e_{14}+e_{15}+y_{13}+y_{12}+e_{12}+e_{11})+e_{14}(y_{15}+y_{11}+e_{15}+e_{11}+y_{14}+y_{13}+e_{13}+e_{12})+e_{15}(y_{15}+y_{14}+e_{14}+e_{13}+y_{11}+y_{12}+e_{11}+e_{12})+e_{21}(y_{21}+e_{23}+y_{22}+e_{22})+e_{22}(y_{22}+e_{21}+y_{23}+e_{23})+e_{23}(y_{21}+e_{21}+y_{23}+e_{22})=956.Mo(D_{6})=\sum_{i=1}^{16}f_{i}|p_{i}-r_{i}|+e_{11}|(x_{11}+x_{15})-(x_{12}+x_{13})|+e_{12}|(x_{12}+x_{11})-(x_{13}+x_{14})|+e_{13}|(x_{13}+x_{12})-(x_{14}+x_{15})|+e_{14}|(x_{15}+x_{11})-(x_{14}+x_{13})|+e_{15}|(x_{11}+x_{12})-(x_{15}+x_{14})|+e_{21}|x_{21}-x_{22}|+e_{22}|x_{22}-x_{23}|+e_{23}|x_{21}-x_{23}|=516.Mo_{e}(D_{6})=\sum_{i=1}^{16}f_{i}|q_{i}-s_{i}|+e_{11}|(y_{11}+y_{15}+e_{15}+e_{14})-(y_{12}+y_{13}+e_{12}+e_{13})|+e_{12}|(y_{12}+y_{11}+e_{11}+e_{15})-(y_{13}+y_{14}+e_{13}+e_{14})|+e_{13}|(y_{14}+y_{15}+e_{14}+e_{15})-(y_{13}+y_{12}+e_{12}+e_{11})|+e_{14}|(y_{15}+y_{11}+e_{15}+e_{11})-(y_{14}+y_{13}+e_{13}+e_{12})|+e_{15}|(y_{15}+y_{14}+e_{14}+e_{13})-(y_{11}+y_{12}+e_{11}+e_{12})|+e_{21}|(y_{21}+e_{23})-(y_{22}+e_{22})|+e_{22}|(y_{22}+e_{21})-(y_{23}+e_{23})|+e_{23}|(y_{21}+e_{21})-(y_{23}+e_{22})|=608.$$

 □

Result 4.7 Let $D_{7}$ be a terizidone compound. Then $W(D_{7})$ = 1297, $W_{e}(D_{7})$ = 1229, $SZ_{v}(D_{7})$ = 1738, $SZ_{e}(D_{7})$ = 1774, $PI(D_{7})$ = 508, $MO(D_{7})$ = 220 and $MO_{e}(D_{7})$ = 248.

**Figure 9:**
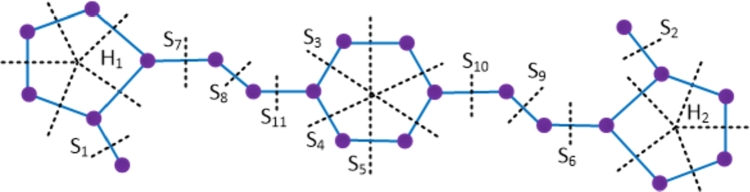
Enumeration of Θ^⁎^-classes in terizidone drug.

Proof With 22 vertices and 24 edges, the molecular graph of terizidone compound has two pentagons and hence terizidone is not a partial cube. Therefore to evaluate the mentioned topological indices, we employ the $\Theta^{⁎}$-partition. Let $\left\{S_{i}:1\leq i\leq 11\}⋃\{H_{1}\}⋃\{H_{2}\right\}$ be the $\Theta^{⁎}$-partition of $D_{7}$. The molecular graph of the terizidone compound along with the $\Theta^{⁎}$ cuts is depicted in Fig. 9. The strength-weighted quantities of the quotient graphs $D_{7}/S_{i}$ and $D_{7}/H_{k}$ are provided in Table 10, Table 11 respectively where we used the vertex weights and strength values as $\left(x_{kj},y_{kj}\right)$ and edge strength values as $e_{kj}$, 1≤j≤5, for the quotient graphs $D_{6}/H_{k}$, k=1,2.

**Table 10:** Strength-weighted values of *D*_7_/*S*_*i*_.

***S***_***i***_	***i* = 1,2**	***i* = 3,4,5**	***i* = 6,7**	***i* = 8,9**	**10**	**11**
***p***_***i***_	1	11	6	7	14	8
***q***_***i***_	0	11	6	7	15	8
***f***_***i***_	1	2	1	1	1	1

**Table 11:** Strength-weighted values of *D*_7_/*H*_*k*_.

k	***x***_***k*****1**_	***x***_***k*****2**_	***x***_***k*****3**_	***x***_***k*****4**_	***x***_***k*****5**_	***y***_***k*****1**_	***y***_***k*****2**_	***y***_***k*****3**_	***y***_***k*****4**_	***y***_***k*****5**_	***e***_***k*****1**_	***e***_***k*****2**_	***e***_***k*****3**_	***e***_***k*****4**_	***e***_***k*****5**_
**1**	1	1	1	17	2	0	0	0	18	1	1	1	1	1	1
**2**	17	2	1	1	1	18	1	0	0	0	1	1	1	1	1

The topological indices are computed using the Eqs. (1) − (7) in Theorem 3.1 as provided below.

$$W(D_{7})=\sum_{i=1}^{11}p_{i}r_{i}+\sum_{k=1}^{2}x_{k1}(x_{k2}+2x_{k3}+2x_{k4}+x_{k5})+x_{k2}(x_{k3}+2x_{k4}+2x_{k5})+x_{k3}(x_{k4}+2x_{k5})+x_{k4}x_{k5}=1297.W_{e}(D_{7})=\sum_{i=1}^{11}q_{i}s_{i}+\sum_{k=1}^{2}[y_{k1}(y_{k2}+2y_{k3}+2y_{k4}+y_{k5})+y_{k2}(y_{k3}+2y_{k4}+2y_{k5})+y_{k3}(y_{k4}+2y_{k5})+y_{k4}y_{k5}+e_{k1}(e_{k3}+e_{k4})+e_{k2}(e_{k4}+e_{k5})+e_{k3}e_{k5}+y_{k1}(e_{k2}+2e_{k3}+e_{k4})+y_{k3}(e_{k4}+2e_{k5}+e_{k1})+y_{k4}(e_{k5}+2e_{k1}+e_{k2})]=1229.Sz_{v}(D_{7})=\sum_{i=1}^{11}f_{i}p_{i}r_{i}+\sum_{k=1}^{2}[e_{k1}(x_{k1}+x_{k5})(x_{k2}+x_{k3})+e_{k2}(x_{k2}+x_{k1})(x_{k3}+x_{k4})+e_{k3}(x_{k3}+x_{k2})(x_{k4}+x_{k5})+e_{k4}(x_{k5}+x_{k1})(x_{k4}+x_{k3})+e_{k5}(x_{k1}+x_{k2})(x_{k5}+x_{k4})]=1738.Sz_{e}(D_{7})=\sum_{i=1}^{11}f_{i}q_{i}s_{i}+\sum_{k=1}^{2}[e_{k1}(y_{k1}+y_{k5}+e_{k5}+e_{k4})(y_{k2}+y_{k3}+e_{k2}+e_{k3})+e_{k2}(y_{k2}+y_{k1}+e_{k1}+e_{k5})(y_{k3}+y_{k4}+e_{k3}+e_{k4})+e_{k3}(y_{k4}+y_{k5}+e_{k2}+e_{k1})(y_{k3}+y_{k2}+e_{k4}+e_{k5})+e_{k4}(y_{k5}+y_{k1}+e_{k5}+e_{k1})(y_{k4}+y_{k3}+e_{k3}+e_{k2})+e_{k5}(y_{k5}+y_{k4}+e_{k1}+e_{k2})(y_{k1}+y_{k2}+e_{k4}+e_{k3})]=1774.PI(D_{7})=\sum_{i=1}^{11}f_{i}(q_{i}+s_{i})+\sum_{k=1}^{2}[e_{k1}(y_{k1}+y_{k5}+e_{k5}+e_{k4}+y_{k2}+y_{k3}+e_{k2}+e_{k3})+e_{k2}(y_{k2}+y_{k1}+e_{k1}+e_{k5}+y_{k3}+y_{k4}+e_{k3}+e_{k4})+e_{k3}(y_{k4}+y_{k5}+e_{k4}+e_{k5}+y_{k3}+y_{k2}+e_{k2}+e_{k1})+e_{k4}(y_{k5}+y_{k1}+e_{k5}+e_{k1}+y_{k4}+y_{k3}+e_{k3}+e_{k2})+e_{k5}(y_{k5}+y_{k4}+e_{k4}+e_{k3}+y_{k1}+y_{k2}+e_{k1}+e_{k2})]=508.Mo(D_{7})=\sum_{i=1}^{11}f_{i}|p_{i}-r_{i}|+\sum_{k=1}^{2}[e_{k1}|(x_{k1}+x_{k5})-(x_{k2}+x_{k3})|+e_{k2}|(x_{k2}+x_{k1})-(x_{k3}+x_{k4})|+e_{k3}|(x_{k3}+x_{k2})-(x_{k4}+x_{k5})|+e_{k4}|(x_{k5}+x_{k1})-(x_{k4}+x_{k3})|+e_{k5}|(x_{k1}+x_{k2})-(x_{k5}+x_{k4})|]=220.Mo_{e}(D_{7})=\sum_{i=1}^{11}f_{i}|q_{i}-s_{i}|+\sum_{k=1}^{2}[e_{k1}|(y_{k1}+y_{k5}+e_{k5}+e_{k4})-(y_{k2}+y_{k3}+e_{k2}+e_{k3})|+e_{k2}|(y_{k2}+y_{k1}+e_{k1}+e_{k5})-(y_{k3}+y_{k4}+e_{k3}+e_{k4})|+e_{k3}|(y_{k4}+y_{k5}+e_{k2}+e_{k1})-(y_{k3}+y_{k2}+e_{k4}+e_{k5})|+e_{k4}|(y_{k5}+y_{k1}+e_{k5}+e_{k1})-(y_{k4}+y_{k3}+e_{k3}+e_{k2})|+e_{k5}|(y_{k1}+y_{k2}+e_{k4}+e_{k3})-(y_{k4}+y_{k5}+e_{k1}+e_{k2})|]=248.$$

 □

Result 4.8 Let $D_{8}$ be an ethambutol compound. Then $W(D_{8})$ = 383, $W_{e}(D_{8})$ = 214, $SZ_{v}(D_{8})$ = 383, $SZ_{e}(D_{8})$ = 214, $PI(D_{8})$ = 156, $MO(D_{8})$ = 100 and $MO_{e}(D_{8})$ = 100.

**Figure 10:**
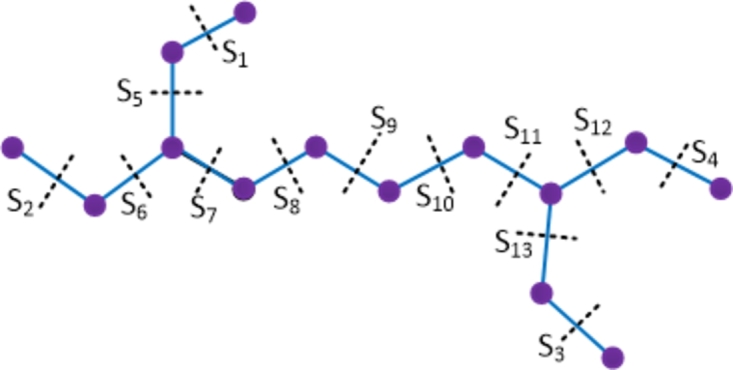
Enumeration of Θ-classes *S*_*i*_ in the ethambutol drug.

Proof The molecular graph of ethambutol has 14 vertices and 13 edges. Let $\{S_{i}$: 1≤i≤13} be the Θ cuts of $D_{8}$ which partition the compound into 2 convex components. The molecular graph of the ethambutol compound along with the Θ cuts is shown in Fig. 10 and strength-weighted quantities are given in Table 12.

**Table 12:** Strength-weighted values of *D*_8_/*S*_*i*_.

***S***_***i***_	**1 ≤ *i* ≤ 4**	***i* = 5,6**	**7**	**8**	**9**	**10**	**11**	***i* = 12,13**
***p***_***i***_	1	2	5	6	7	8	9	12
***q***_***i***_	0	1	4	5	6	7	8	11
***f***_***i***_	1	1	1	1	1	1	1	1

The mentioned topological indices can be obtained by using the simple mathematical calculations underlying the cut method. □

Result 4.9 Let $D_{9}$ be an ethionamide compound. Then $W(D_{9})$ = 156, $W_{e}(D_{9})$ = 79, $SZ_{v}(D_{9})$ = 240, $SZ_{e}(D_{9})$ = 133, $PI(D_{9})$ = 104, $MO(D_{9})$ = 53 and $MO_{e}(D_{9})$ = 58.

**Figure 11:**
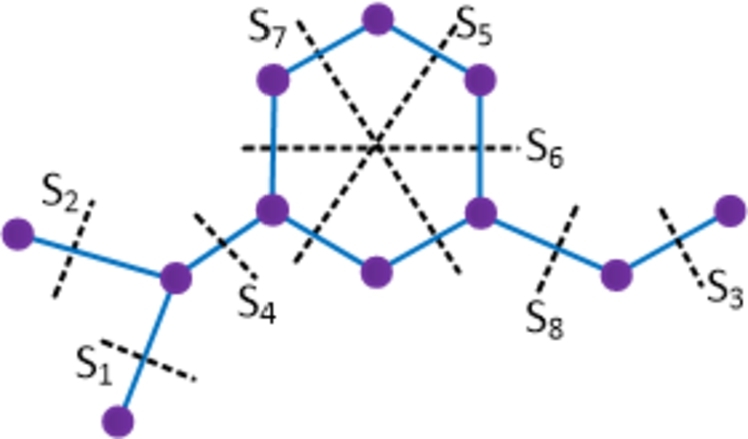
Enumeration of Θ-classes *S*_*i*_ in the ethionamide drug.

Proof The chemical graph of ethionamide has 11 vertices and 11 edges. Let $\{S_{i}$: 1≤i≤8} be the Θ cuts of $D_{9}$ which partition the compound into 2 convex components. The molecular graph of the ethionamide compound along with the Θ cuts is shown in Fig. 11 and strength-weighted quantities are given in Table 13.

**Table 13:** Strength-weighted values of *D*_9_/*S*_*i*_.

***S***_***i***_	***i* = 1,2,3**	**4**	**5**	**6**	**7**	**8**
***p***_***i***_	1	3	5	8	6	9
***q***_***i***_	0	2	4	7	5	9
***f***_***i***_	1	1	2	2	2	1

The mentioned topological indices can be obtained by using the simple mathematical calculations underlying the cut method. □

Result 4.10 Let $D_{10}$ be an isoniazid compound. Then $W(D_{10})$ = 121, $W_{e}(D_{10})$ = 62, $SZ_{v}(D_{10})$ = 184, $SZ_{e}(D_{10})$ = 98, $PI(D_{10})$ = 84, $MO(D_{10})$ = 48 and $MO_{e}(D_{10})$ = 52.

**Figure 12:**
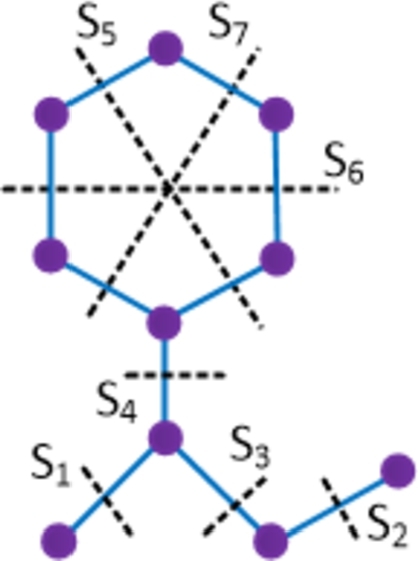
Enumeration of Θ-classes *S*_*i*_ in the isoniazid drug.

Proof The chemical graph of isoniazid has 10 vertices and 10 edges. Let $\{S_{i}$: 1≤i≤7} be the Θ cuts of $D_{10}$ which partition the compound into 2 convex components. The molecular graph of the isoniazid compound along with the Θ cuts is depicted in Fig. 12 and strength-weighted quantities are presented in Table 14. The numerical quantities can be obtained by simple mathematical calculations. □

**Table 14:** Strength-weighted values of *D*_10_/*S*_*i*_.

***S***_***i***_	***i* = 1,2**	**3**	**4**	***i* = 5,6**	**7**
***p***_***i***_	1	8	4	7	3
***q***_***i***_	0	8	3	6	2
***f***_***i***_	1	1	1	2	2

Result 4.11 Let $D_{11}$ be a levofloxacin compound. Then $W(D_{11})$ = 1484, $W_{e}(D_{11})$ = 1255, $SZ_{v}(D_{11})$ = 3098, $SZ_{e}(D_{11})$ = 2777, $PI(D_{11})$ = 782, $MO(D_{11})$ = 394 and $MO_{e}(D_{11})$ = 468.

**Figure 13:**
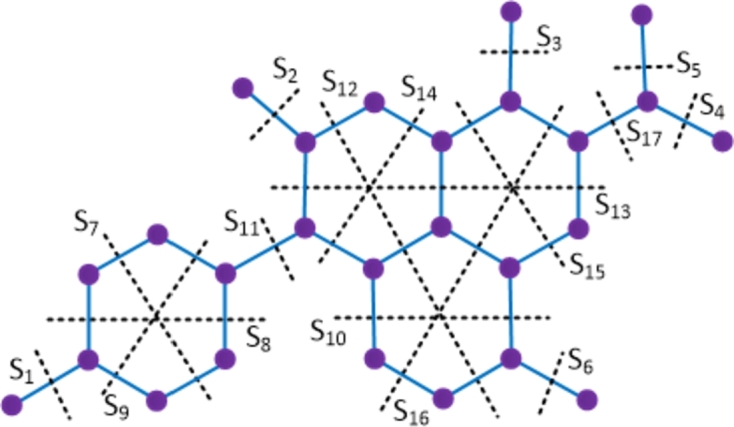
Enumeration of Θ-classes *S*_*i*_ in the levofloxacin drug.

Proof The molecular graph of levofloxacin has 26 vertices and 29 edges. Let $\{S_{i}$: 1≤i≤17} be the Θ cuts of $D_{11}$ which partition the compound into 2 convex components. The molecular graph of the levofloxacin compound along with the Θ cuts is depicted in Fig. 13 and strength-weighted quantities are presented in Table 15. By performing routine calculations, we obtain the required indices. □

**Table 15:** Strength-weighted values of *D*_11_/*S*_*i*_.

***S***_***i***_	**1 ≤ *i* ≤ 6**	**7 ≤ *i* ≤ 10**	**11**	**12**	**13**	**14**	**15**	**16**	**17**
***p***_***i***_	1	4	7	13	16	11	19	9	3
***q***_***i***_	0	3	7	13	17	11	21	8	2
***f***_***i***_	1	2	1	3	3	2	2	3	1

Result 4.12 Let $D_{12}$ be a *p*-aminosalicylic acid compound. Then $W(D_{12})$ = 150, $W_{e}(D_{12})$ = 72, $SZ_{v}(D_{12})$ = 236, $SZ_{e}(D_{12})$ = 128, $PI(D_{12})$ = 104, $MO(D_{12})$ = 55 and $MO_{e}(D_{12})$ = 60.

**Figure 14:**
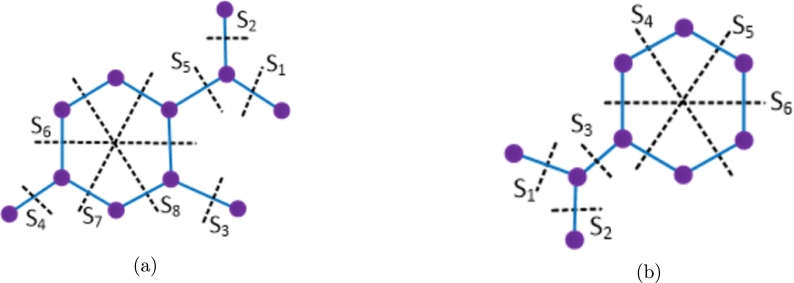
(a) Enumeration of Θ-classes *S*_*i*_ in *p*-aminosalicylic acid, (b) Enumeration of Θ-classes *S*_*i*_ in pyrazinamide.

Proof The molecular graph of *p*-aminosalicylic acid has 11 vertices and 11 edges. Let $\{S_{i}$: 1≤i≤8} be the Θ cuts of $D_{12}$. The molecular graph of the *p*-aminosalicylic acid compound along with the Θ cuts is depicted in Fig. 14(a) and the topological indices are obtained by using the strength-weighted quantities presented in Table 16. □

**Table 16:** Strength-weighted values of *D*_12_/*S*_*i*_.

***S***_***i***_	**1 ≤ *i* ≤ 4**	**5**	**6**	***i* = 7,8**
***p***_***i***_	1	3	5	7
***q***_***i***_	0	2	4	6
***f***_***i***_	1	1	2	2

Result 4.13 Let $D_{13}$ be a pyrazinamide compound. Then $W(D_{13})$ = 88, $W_{e}(D_{13})$ = 42, $SZ_{v}(D_{13})$ = 142, $SZ_{e}(D_{13})$ = 72, $PI(D_{13})$ = 66, $MO(D_{13})$ = 35 and $MO_{e}(D_{13})$ = 38.

Proof The chemical graph of pyrazinamide has 9 vertices and 9 edges. Let $\{S_{i}$: 1≤i≤6} be the Θ cuts of $D_{12}$. The molecular graph of the pyrazinamide compound along with the Θ cuts is depicted in Fig. 14(b) and strength-weighted quantities are presented in Table 17. By making mathematical calculations, we get the required result. □

**Table 17:** Strength-weighted values of *D*_13_/*S*_*i*_.

***S***_***i***_	***i* = 1,2**	**3**	***i* = 4,5,6**
***p***_***i***_	1	3	3
***q***_***i***_	0	2	2
***f***_***i***_	1	1	2

## 5 Discussion

Quantitative structure-property relationships (QSPR) are mathematical models that use graph theoretical techniques to obtain the structural properties of a molecule which in turn help us to predict their macroscopic properties. These models were initially employed in biology and toxicology but recently they are being used more frequently to predict physico-chemical properties of chemical compounds. They use multiple linear regression to estimate the correlation between the critical properties of the target compound and the molecular descriptors using proper coefficients [45], [46], [47], [48], [49], [50]. QSPR models are widely used in chemometrics, pharmacodynamics, pharmacokinetics, toxicology, and many other fields as a common and effective research strategy. They reduce time and cost, rationally predict biological, pharmaceutical, physical and chemical activities and properties and also help the experimental scientists by providing useful data to shed light on the mechanism of action for biological activities of the desired compound. They have wide range of applications in biology, agriculture, medicine, physical and organic chemistry and material science. They are successfully being utilized for predicting absorption, distribution, metabolism, excretion and toxicity. Further, they are used to predict flash point, energy, and many such properties of chemical compounds which can be found in various literatures [51], [52], [53], [54], [55], [56], [57], [58], [59], [60], [61].

**Figure 15:**
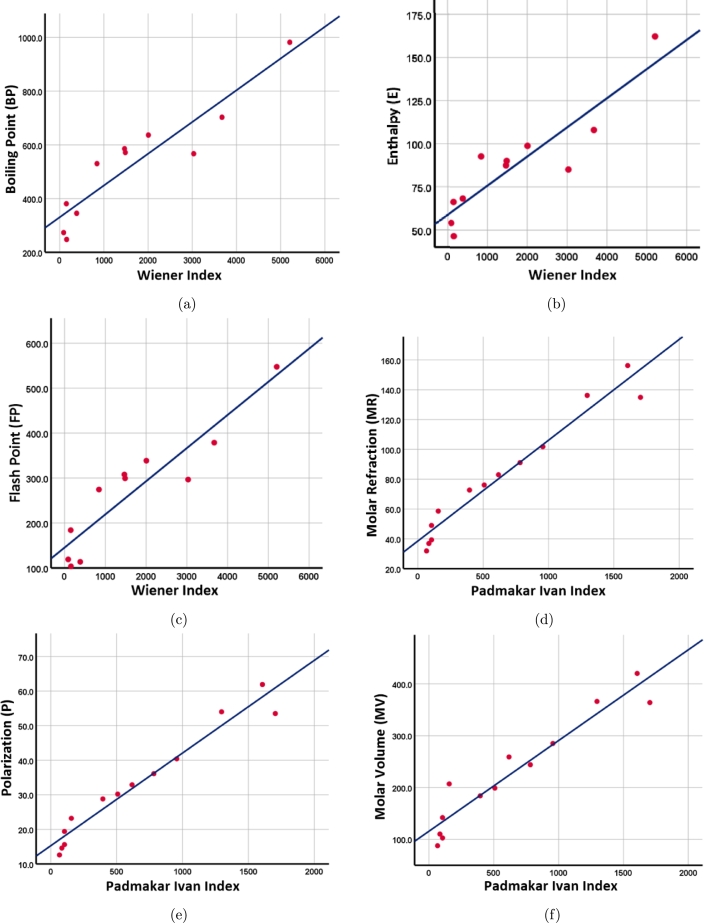
Regression lines (a) Wiener with boiling point (b) Wiener with enthalpy (c) Wiener with flash point (d) PI with molar refraction (e) PI with polarization (f) PI with molar volume.

We consider seven topological indices that are distance-based for modeling the attributes such as boiling point (BP), enthalpy (E), flash point (FP), molar refraction (MR), polarizability (P), molar volume (MV) of 13 drugs. The values of the several physicochemical properties obtained from PubChem (https://pubchem.ncbi.nlm.nih.gov) and ChemSpider (http://www.chemspider.com) are presented in Table 18. The correlation coefficient between the distance based molecular descriptors and the physicochemical properties of 13 anti-TB drugs is enlisted in Table 19. In this paper, the linear regression model PP=A+B[TI] is used to correlate the physicochemical property (PP) of various anti-TB drugs with topological index (TI). Among the linear regression models, the most suitable fitting models for the physicochemical properties of various drugs used in the treatment of tuberculosis are listed in Table 20 and their regression lines are shown in Fig. 15(a–f).

**Table 18:** Physicochemical properties of the anti-TB drugs.

**Compounds**	**BP**	**E**	**FP**	**MR**	**P**	**MV**
**Amikacin**	981.8	162.2	547.6	134.9	53.5	363.9
**Bedaquiline**	702.7	108	378.8	156.2	61.9	420.1
**Clofazimine**	566.9	85.1	296.7	136.2	54	366.1
**Ethambutol**	345.3	68.3	113.7	58.6	23.2	207
**Ethionamide**	247.9	46.5	103.7	49	19.4	142
**Imipenem-cilastatin**	530.2	92.7	274.5	72.7	28.8	183.9
**Isoniazid**	-	-	-	36.9	14.6	110.2
**Levofloxacin**	571.5	90.1	299.4	91.1	36.1	244
**Linezolid**	585.5	87.5	307.9	83	32.9	259
**Moxifloxacin**	636.4	98.8	338.7	101.8	40.4	285
**p-aminosalicylic acid**	380.8	66.3	184.1	39.3	15.6	102.7
**Pyrazinamide**	273.3	54.1	119.1	31.9	12.6	87.7
**Terizidone**	-	-	-	76.1	30.2	198.9

**Table 19:** Correlation between topological indices and physicochemical properties of anti-TB drugs.

**TI**	**BP**	**E**	**FP**	**MR**	**P**	**MV**
***W***	**0.929**	**0.903**	**0.922**	0.927	0.927	0.909
***We***	0.913	0.874	0.909	0.946	0.946	0.927
***S******z***_***v***_	0.897	0.849	0.895	0.954	0.954	0.933
***S******z***_***e***_	0.881	0.823	0.882	0.961	0.962	0.940
***PI***	0.902	0.846	0.902	**0.976**	**0.976**	**0.956**
***MO***	0.898	0.862	0.893	0.950	0.950	0.932
***M******O***_***e***_	0.891	0.848	0.889	0.960	0.960	0.941

**Table 20:** The most suitable fitting linear regression models for the physicochemical properties of various drugs used in the treatment of tuberculosis.

**Model**	***R***^**2**^	**Adjusted*****R***^**2**^	**Std. Error**	***F*-*value***	***p*-*value***
*BP* = 330.756 + 0.118*W*	0.862	0.847	83.3060	56.315	0.00003700
*E* = 58.858 + 0.017*W*	0.816	0.796	14.1309	39.985	0.00013700
*FP* = 145.575 + 0.074*W*	0.851	0.834	54.4604	51.313	0.00005300
*MR* = 38.579 + 0.068*PI*	0.953	0.949	9.2058	223.140	0.00000001
*P* = 15.277 + 0.027*PI*	0.953	0.949	3.6451	223.984	0.00000001
*MV* = 115.757 + 0.175*PI*	0.913	0.905	33.1220	115.515	0.00000030

From Table 20, we observe that the Wiener and PI indices are dominating over the other indices such as Szeged and Mostar for predicting the physicochemical properties of drugs. In addition, we see that the *p*-values for the proposed models are below 0.05, alongside larger *F*-values, thus providing the clear significance of these proposed models. Moreover, the Szeged and Mostar indices are computed based on the closeness sets of each bond in the drug compounds by taking the mathematical operations of multiplication and absolute difference respectively, compared to the addition in the case of the PI index and hence, the Szeged and Mostar indices do not exhibit superior performance, however these indices may be helpful for analyzing the structural properties of large complex structures.

## 6 Conclusion

The distance-based topological indices of anti-tuberculosis drugs are computed, and QSPR analysis of the indices with the chosen properties reveals that the Wiener index correlates well with boiling point, enthalpy, and flash point, whereas the Padmakar-Ivan index correlates well with molar refraction, polarisation, and molar volume. Moreover, if we compare our proposed models with the earlier existing models based on degree [29], our correlations are high in the cases of boiling point, enthalpy, and flash point, while in the cases of molar refraction, polarization, and molar volume, they are marginally less; however, we believe that the predicting capacity of distance based models is more reliable for large compounds. Hence, the findings of this study could be crucial in the development of new tuberculosis drugs and vaccines. Finally, we would like to highlight that a significant portion of recent research [62], [63], [64], [65], [66] in QSPR modeling relies on degree-based topological indices and an intriguing avenue for further exploration involves the development of distance-dependent models specifically tailored for COVID-19 drug molecules.

## CRediT authorship contribution statement

**Micheal Arockiaraj:** Formal analysis, Conceptualization. **Joseph H. Campena:** Writing – review & editing, Visualization, Investigation, Funding acquisition. **A. Berin Greeni:** Methodology. **Muhammad Usman Ghani:** Resources, Project administration. **S. Gajavalli:** Investigation, Data curation. **Fairouz Tchier:** Methodology, Investigation, Funding acquisition, Formal analysis. **Ahmad Zubair Jan:** Writing – original draft, Validation, Software, Conceptualization.

## Declaration of Competing Interest

The authors declare that there is no conflict of interest regarding the publication of this article. All the authors of the paper have agreed to submit this paper.

## Data Availability

No data was used for the research described in the article.
